# Sexually transmitted infection screening to prevent adverse birth and newborn outcomes: study protocol for a randomized-controlled hybrid-effectiveness trial

**DOI:** 10.1186/s13063-022-06400-y

**Published:** 2022-05-24

**Authors:** Andrew Medina-Marino, Susan Cleary, Christina A. Muzny, Christopher Taylor, Ashutosh Tamhane, Phuti Ngwepe, Charl Bezuidenhout, Shelley N. Facente, Koleka Mlisana, Remco P. H. Peters, Jeffrey D. Klausner

**Affiliations:** 1 Department of Psychiatry, Perelman School of Medicine, University of Pennsylvania, 3535 Market St, 3rdFloor, Philadelphia, PA 19104 USA; 2 Division of Men’s Health, Desmond Tutu HIV Centre, University of Cape Town, Cape Town, South Africa; 3 Health Economics Unit, School of Public Health and Family Medicine, University of Cape Town, Cape Town, South Africa; 4 Division of Infectious Diseases, University of Alabama at Birmingham, Birmingham, USA; 5 Department of Microbiology, Immunology, and Parasitology, Louisiana State University Health Sciences Center, New Orleans, USA; 6 Microbial Genomics Resource Group, Louisiana State University Health Sciences Center, New Orleans, USA; 7 Department of Medicine, University of Alabama at Birmingham, Birmingham, USA; 8 Department of Statistical Sciences, University of Cape Town, Cape Town, South Africa; 9 Research Unit, Foundation for Professional Development, East London, South Africa; 10 School of Public Health, Boston University, Boston, USA; 11 School of Public Health, University of California, Berkeley, Berkeley USA; 12 Department of Medical Microbiology, School of Laboratory Medicine and Medical Sciences, University of KwaZulu-Natal, Mayville, South Africa; 13 Academic Affairs, Research and Quality Assurance, National Health Laboratory Service, Johannesburg, South Africa; 14 Department of Medical Microbiology, University of Pretoria, Pretoria, South Africa; 15 Departments of Population and Public Health Sciences and Medicine, Keck School of Medicine, University of Southern California, Los Angeles, USA

**Keywords:** Sexually transmitted infections, STIs; Pregnancy, Preterm birth, Low birth weight, Antenatal care, STI screening, Syndromic management, Cost-effectiveness

## Abstract

**Background:**

Sexually transmitted infections (STIs) during pregnancy are associated with adverse birth outcomes, including preterm birth, low birth weight, perinatal death, and congenital infections such as increased mother-to-child HIV transmission. Prevalence of STIs among pregnant women in South Africa remains high, with most women being asymptomatic for their infection(s). Unfortunately, most STIs remain undetected and untreated due to standard practice syndromic management in accordance with World Health Organization (WHO) guidelines. Although lab-based and point-of-care molecular tests are available, optimal screening strategies during pregnancy, their health impact, and cost-effectiveness are unknown.

**Methods:**

We will implement a 3-arm (1:1:1) type-1 hybrid effectiveness-implementation randomized-controlled trial (RCT). We will enroll 2500 pregnant women attending their first antenatal care (ANC) visit for their current pregnancy at participating health facilities in Buffalo City Metro District, Eastern Cape Province, South Africa. Participants allocated to arms 1 and 2 (intervention) will receive GeneXpert® point-of-care diagnostic testing for *Neisseria gonorrhoeae*, *Chlamydia trachomatis*, and *Trichomonas vaginalis*, with same-day treatment for detected infection(s). Arm 1 will additionally receive a test-of-cure 3 weeks post-treatment, while Arm 2 will receive a repeat test at 30–34 weeks’ gestation. Those allocated to Arm 3 will receive syndromic management (standard-of-care). The RE-AIM framework will be used to guide collection of implementation indicators to inform potential future scale up. Primary outcome measures include (1) frequency of adverse birth outcomes among study arms, defined by a composite measure of low birth weight and pre-term delivery, and (2) change in STI prevalence between baseline and birth outcome among intervention arms and compared to standard-of-care. Estimates and comparative costs of the different screening strategies relative to standard-of-care and the costs of managing adverse birth outcomes will be calculated. Cost-effectiveness will be assessed per STI and disability-adjusted life year averted.

**Discussion:**

This trial is the first RCT designed to identify optimal, cost-effective screening strategies that decrease the burden of STIs during pregnancy and reduce adverse birth outcomes. Demonstrating the impact of diagnostic screening and treatment, compared to syndromic management, on birth outcomes will provide critical evidence to inform changes to WHO guidelines for syndromic management of STIs during pregnancy.

**Trial registration:**

ClinicalTrials.gov NCT04446611. Registered on 25 June 2020.

## Trial registration data

**Table:** 

Data category	Information
Primary registry and trial identifying number	ClinicalTrials.gov NCT04446611
Date of registration in primary registry	25 June 2020
Secondary identifying numbers	R01AI149339
Source(s) of monetary or material support	National Institutes of Health, National Institute of Allergy and Infectious Diseases (NIAID)
Primary sponsor	National Institutes of Health
Contact for public queries	Andrew Medina-Marino, PhD [andrewmedinamarino@gmail.com] Jeffrey Klausner, MD, MPH [jdklausner@med.usc.edu]
Contact for scientific queries	Andrew Medina-Marino, PhD [andrewmedinamarino@gmail.com] Jeffrey Klausner, MD, MPH [jdklausner@med.usc.edu]
Public title	Clinical Study of STI Screening to Prevent Adverse Birth and New-born Outcomes
Scientific title	R01 STI Screening and Microbiome Study: Clinical Study of STI Screening to Prevent Adverse Birth and New-born Outcomes
Countries of recruitment	South Africa
Health condition(s) or problem(s) studied	*Neisseria gonorrhoeae* (NG), *Chlamydia trachomatis* (CT) and *Trichomonas vaginalis* (TV) infection during pregnancy
Intervention(s)	Treatment Group 1: Single point-of-care molecular diagnostic screening and treatment for CT, NG, and TV at first antenatal care visit and infection-specific test-of-cure 3 weeks post-treatment. Women with a positive test-of-cure will be re-treated
	Treatment Group 2: Repeated point-of-care molecular diagnostic screening and treatment for CT, NG, and TV at first antenatal care visit and at week 30–34 gestation. No test-of-cure will be conducted for women with positive test results; however, additional treatment will be provided to women with persistent/recurrent vaginal discharge
	Control Group: Syndromic management at every antenatal care visit (standard of care)
Key inclusion and exclusion criteria	Ages eligible for study: ≥ 18 years Sexes eligible for study: female Accepts healthy volunteers: yes
	Inclusion criteria: adult patient (≥ 18 years), attending first antenatal care visit for current pregnancy, gestational age < 27 weeks, agreeing to nurse-collected specimens, and intent to deliver in one of the participating study clinics
	Exclusion criteria: planning to relocate during pregnancy or deliver in a non-participating study clinic, currently participating in another antenatal care/HIV study, and/or ultrasound-confirmed gestational age > 26 weeks 6 days at first antenatal care visit
Study type	Interventional
	Allocation: randomizations are allocated in blocks of 15 with a 1:1:1 randomization into the 3 study arms across the participating facilities. Parallel assignment masking: double blind (participant, study staff), though participation arm is non-blinded
	Primary purpose: screening
	Phase III
Date of first enrollment	29 March 2021
Target sample size	2500
Recruitment status	Recruiting
Primary outcome(s)	Frequency of adverse birth outcomes at delivery among study arms, as defined by a composite measure of low birth weight and pre-term delivery; Change in STI prevalence between baseline (first antenatal care visit) and birth outcome among study arms [Time frame: Change in STI between baseline (< 26 weeks gestation) and delivery (approximately 38–42 gestation) up to 2 weeks post-delivery], [Time frame: through study completion, an average of 1 month]
Key secondary outcomes	Prevalence and risk factors for CT, NG, and TV in neonates, controlling for HIV status; the prevalence and risk factors for STI at birth outcome among mothers; factors associated with STIs at first antenatal care visit; incremental cost per STI and DALY averted

## Background

Sexually transmitted infections (STIs) during pregnancy are associated with multiple adverse birth outcomes such as preterm birth, low birth weight, perinatal death, and congenital infections including increased mother-to-child HIV transmission [1–12]. In South Africa, HIV and STIs among pregnant women are a major problem, with an estimated 30.7% of women seeking antenatal care (ANC) found to be living with HIV [13], compounded by high rates of STIs in South African women of reproductive age [14–16].

Though STIs are common in pregnant women globally, current syndromic management guidelines from both the World Health Organization (WHO) and South Africa continue to result in the majority of STIs (most of which are asymptomatic) remaining undetected and untreated during pregnancy [17–26]. Syndromic management involves treating STIs based on an algorithm of common symptoms, and WHO recommends syndromic management of STIs in resource-limited settings due to low cost and the unavailability of appropriate laboratory infrastructure for diagnosis-based treatment [27, 28]. Major limitations of syndromic management include (1) non-determination of infectious etiologies; (2) limited specificity, especially during pregnancy, of “symptoms” algorithms; and (3) inappropriate treatment or over-treatment [25, 29]. Alternatives for simple, point-of-care STI diagnosis are desperately needed in low-and-middle-income countries to allow for improved treatment of pregnant women living with STIs, ultimately reducing adverse birth outcomes.

We previously conducted a study to examine the acceptability and feasibility of integrating point-of-care molecular diagnostic screening for *Chlamydia trachomatis* (CT), *Neisseria gonorrhoeae* (NG), and *Trichomonas vaginalis* (TV) into ANC services for HIV-infected pregnant women in South Africa [30–33]. We found diagnostic screening and immediate treatment during ANC to be highly acceptable and feasible [26]; 97.8% agreed to be tested and > 93% received same-day treatment. Of 430 women screened, 41% had an STI, of which 65% were asymptomatic [26]. Our intervention decreased prevalent STIs at time of delivery by > 50% compared to women who received standard-of-care syndromic management.

Though acceptable, feasible, and effective, our previous study had limitations. First, we detected an STI incidence rate of 15 infections per 100 women-years among pregnant women in our study, suggesting a single diagnostic screening with appropriate treatment at ANC enrollment may not optimally decrease STIs at time of delivery [34]. Second, our study was underpowered to demonstrate an effect on birth outcomes [35]. Third, we found a persistent 26.5% STI positivity at the test-of-cure visit three weeks after the initial baseline visit and targeted treatment [36]. Though studies suggest that untreated partners are the primary cause of persistent STI positivity in women, in our study among women with a treated partner, persistent STIs were still high [36, 37]. Consequently, biological factors that increase the risk for STI persistence must be further investigated. Lastly, we did not conduct a cost-effectiveness analysis, which is urgently needed to inform policy design [38]. The South African National Strategic Plan for HIV, TB, and STIs 2017–2022 [39] includes recommendations for the detection and treatment of STIs, including through point-of-care testing. However, to date, no South African study exists to inform those cost and budget efforts. This trial has been designed to overcome the limitations of the previous pilot study, providing necessary information to inform policy decisions in South Africa and other low-and-middle income countries, as well as WHO recommendations for the management of STIs during pregnancy.

## Methods/design

### Study aims

Our RCT has two main study aims: (1) to evaluate different STI screening strategies to decrease the burden of CT, NG, and TV among pregnant women and reduce STI-related adverse birth outcomes and (2) to evaluate the cost-effectiveness of different STI screening approaches to inform guideline and policy development.

### Study setting and design

STI screening and/or treatment for CT, NG, and TV will be offered to 2500 women age 18 years and older who present for first ANC services in one of three ANC clinics in Buffalo City Metro Health District, Eastern Cape Province, South Africa. Participants will be enrolled in an effectiveness-implementation hybrid type 1 three-arm (1:1:1) randomized-controlled trial, with the following arms:

- *Arm 1—treatment group (n* = *833)*: Participants will receive single point-of-care molecular diagnostic screening and treatment for CT, NG, and TV at their first ANC visit, and infection-specific test-of-cure 3 weeks post-treatment. Women with a positive test-of-cure will be re-treated. As CT/NG is a combination Xpert test, at test-of-cure, participants may also be diagnosed with an incident infection of the other bacterium, which would be treated and managed accordingly.
- *Arm 2—treatment group (n* = *833)*: Participants will receive repeat point-of-care molecular diagnostic screening and treatment for CT, NG, and TV, at both the first ANC visit and the week 30–34 visit. No test-of-cure will be conducted for women with positive test results; however, additional treatment will be provided to women with persistent/recurrent vaginal discharge.
- *Arm 3—control group (n* = *834)*: Participants will receive syndromic management (standard of care) at every ANC visit, per current South African National Guidelines [40, 41].

In all arms, women are followed until the postnatal visit and infants through the 6-week infant immunization visit to collect pregnancy and birth-outcome data as well as neonatal health outcomes (morbidities or mortality). Depending on the randomization arm, participants are scheduled to be seen various times throughout pregnancy by the study team; ANC visits are conducted in line with national policy. All post-partum mothers and infants are asked to be seen at the first post-delivery clinic visit. Table 1 outlines the STI testing time-points for the different study arms.

**Table 1:** STI testing schedule per randomization arm

Clinic visit	Participant	Specimen collected	CT, NG, and TV testing
First ANC visit	All pregnant women	Vaginal smear Vaginal swabs	Arms 1 and 2 only
Test-of-cure 3 weeks post treatment	Arm 1 only	Vaginal smear Vaginal swabs	Arm 1 only
30–34 weeks’ gestation	Arm 2 only	Vaginal smear Vaginal swabs	Arm 2 only
First post-delivery clinic visit	All post-partum mothers	Vaginal swabs	All post-partum mothers
First post-delivery clinic visit	All infants	Nasopharyngeal swabs Conjunctival swabs	All infants^a^

^a^Post-delivery infant swabs will only be tested if the maternal swab is positive

For Aim 2, combined top down and bottom up methodologies will establish the societal costs of the different STI screening strategies relative to control including the costs of managing adverse birth outcomes. Decision analytic modeling will estimate the incremental cost per STI and disability-adjusted life year (DALY) averted.

### Recruitment and enrollment

Following standard HIV testing per South African National Guidelines, all pregnant women presenting for ANC services at one of the study clinics will be screened for eligibility by a clinic-based STI test counselor or a research nurse working for the study. Study staff will read all interested women a brief study description and use an electronically administered eligibility screening tool based in REDCap [42], which allows the system to flag individuals as eligible or ineligible in real-time, per the following:

#### Inclusion criteria

To be eligible for participation in the study, women must meet the following criteria: (1) age ≥ 18 years, (2) currently pregnant, (3) attending the first ANC visit for current pregnancy, (4) gestational age < 27 weeks*, (5) agreeing to nurse-collected vaginal specimens, and (6) intent to deliver in one of the three participating study clinics.

#### Exclusion criteria

Women are excluded from the study if they meet any of the following criteria: (1) planning to relocate during pregnancy or deliver in a clinic not participating in the study, (2) currently participating in another ANC/HIV study, and (3) ultrasound confirmed > 26 weeks 6 days gestation at first ANC. All women < 27-week gestation calculated by last normal menstrual period undergo an ultrasound to confirm gestational age and eligibility; per good clinical practice, women are asked to urinate prior to ultrasound for gestational age determination. Even though WHO and South African guidelines recommend that pregnant women attend their first ANC visit before 20 weeks to achieve optimal pregnancy outcomes, a considerable proportion of pregnant women in South Africa only come for their first ANC visit after 20 weeks’ gestation [43, 44]. To be able to include stillbirth as an outcome, we selected a cut-off of 26 weeks 6 days’ gestational age at first ANC as an inclusion/exclusion criterion, in line with other current studies [45].

**Fig. 1:**
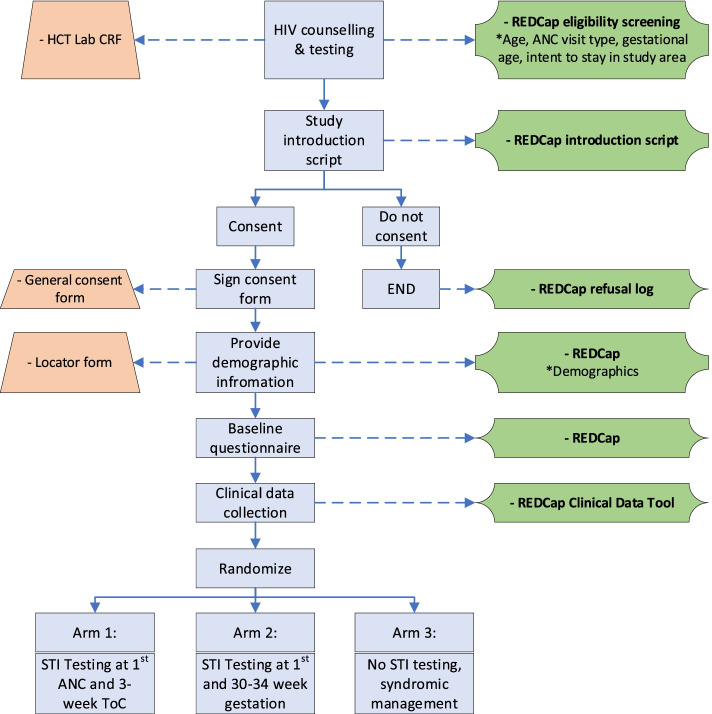
Participant recruitment and enrollment flow

Interested eligible women are then read aloud the study consent form by study staff in their preferred language (English or isiXhosa) and are invited to participate. Study staff record reasons for ineligibility/refusal, if provided. Those providing written informed consent are enrolled and asked to provide demographic information and complete a baseline questionnaire, before being randomized into one of the 3 study arms (see Fig. 1). Randomizations are allocated in blocks of 15 with a 1:1:1 randomization into the 3 study arms. Study staff are blinded to the randomization blocks and status. Automated randomization is performed by the randomization module on REDCap, ensuring allocation concealment. Once study arm has been assigned, study staff inform the participant of their assignment and upcoming study procedures, at which time both study staff and participants are unblinded due to the open nature of study activities.

### Retention and adherence

To ensure participant retention, those providing informed consent will be asked to provide detailed contact information (e.g., phone numbers and home address for self, family, friend/neighbor). To develop and maintain a strong relationship with participants, study staff will conduct welcome phone calls within 3 days of enrollment and check in with participants during regular ANC clinic visits or monthly antiretroviral therapy pickup for those with HIV. Appointment reminders will be sent via SMS, and a telephonic reminder will be done prior to the visit. Women will also be given an appointment card as a reminder for future visits which will also include the research nurses’ contact information.

Participant charts will be flagged so that clinic staff will know to notify study staff on date of delivery. Seven days post-delivery, study staff will contact participants not yet attending a first postnatal clinic visit to schedule an outcomes interview. We will make up to seven attempts to follow up with participants via text/phone call/home visits. When the participants return to report their pregnancy outcome, we will provide a reimbursement of a R100 ($1USD = R14.5) gift voucher to use at local stores.

### Sample size and power calculations

Aim 1 analyses will explore intervention effects on reducing probabilities for adverse birth outcomes and STI prevalence at time of delivery. Based on a total sample size of 2500 participants (833–834 participants in each study arm), calculations show that we will have at least 80% power to detect study arm absolute differences of approximately 10% or larger in the frequency of adverse birth outcomes. We conducted two sets of calculations. (1) Calculations for the probability of an adverse birth event were conducted in PASS 2008 software (https://www.ncss.com/) for differences in proportions at a single time point (i.e., at birth). Calculations were run for a range of base rates ranging from 30 to 50%; this is in line with base rates from preliminary data (~ 40%). (2) We calculated changes in STI prevalence based on two time points (i.e., first ANC visit and birth) and conducted simulation studies in two steps. First, we simulated STI data from a binomial distribution with parameter values based on preliminary data. Preliminary results gave pregnancy STI rates around 40%; simulations used a range of pregnancy STI rates from 30 to 50%. Based on preliminary data, we anticipate that the intervention will reduce STI rates by 20% (absolute). We assumed an attrition rate of 15%.

### Data collection

#### Aim 1

At the time of enrollment, trained study staff administer, in a private space, a baseline REDCap questionnaire to all participants in their preferred language. The questionnaire is adapted, in part, from measures used by our team in previous and current STI screening and maternal-child health studies, or from other studied documented in the literature. The questionnaire is expected to take no more than 45 min and includes participant: (a) demographics, socio-economic status, and patient costs of seeking care; (b) obstetric, gynecological, and sexual health history; (c) sexual behaviors, risk factors, and perceived risk for HIV/STI acquisition before and during pregnancy; (d) partner characteristics and HIV status; and (e) previous history of STIs.

Staff abstract additional clinical history from each participant’s maternity case record, including HIV status, date of diagnosis, and immunological characteristics associated with HIV infection (e.g., CD4 T-cell level, HIV viral load, type of antiretroviral therapy, antiretroviral therapy use/duration). The maternity case record is used from the day of first ANC consultation to record clinical information throughout the duration of the pregnancy. Staff verify self-reported and medical record-abstracted HIV-related information.

Participants in Arm 3 (receiving syndromic management) are also seen by the study nurse at first ANC visit. As all participants are seen by clinic staff for their follow-up ANC appointments, any syndromic management given during ANC is indicated on the ANC charts by the clinic nurses. Study staff maintains a close relationship with clinic staff to extract any management information and treatment given for STIs during any ANC visit. One of the two STI counselors at the study site is dedicated to assist with data extraction activities, including data extraction ANC charts to determine syndromic management outcomes for participants.

**Fig. 2:**
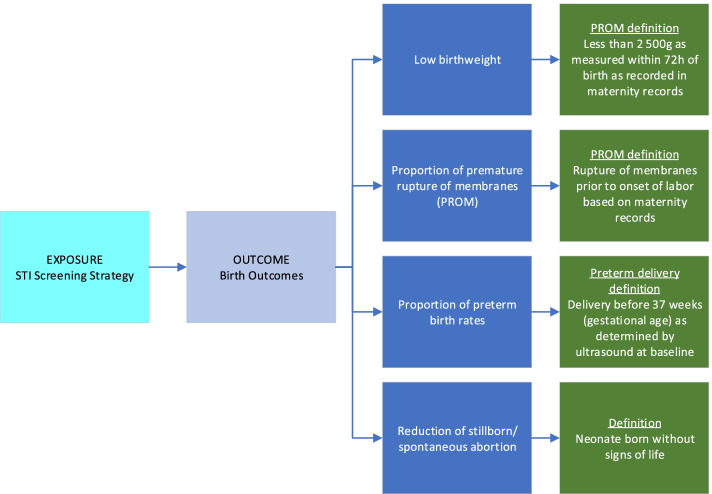
Birth outcomes

Data on pregnancy and birth outcomes (Fig. 2) are collected on all study participants via abstraction of labor/postnatal delivery registers and face-to-face interviews with participants during the first postnatal clinic visit. All clinical data relating to labor, delivery, and birth/neonatal outcomes are recorded on a discharge summary; women are given a copy of discharge summaries when they leave the clinic (a carbon copy is kept in the labor ward). Additional data are abstracted from the infant health record, known as the Road-to-Health card, which is issued to all infants born in South African facilities. Staff collect information on fetal loss, preterm labor, preterm birth, birth weight, the calculated small-for-gestational-age status, and infant mortality. Information on potential confounding variables such as maternal history of chronic illness (e.g., hypertension, diabetes), other infections during pregnancy (e.g., urinary tract infections, syphilis), antibiotic use during pregnancy, and pregnancy complications (e.g., premature rupture of membranes, maternal fever, chorioamnionitis, and pre-eclampsia) is also collected. HIV PCR results from routine at-birth testing of HIV-exposed infants are collected via clinical records and verified using the National Health Laboratory Service LabTrack system.

At the routine 6-week immunization visit, data are collected on neonatal health outcomes and morbidities (or mortality) (i.e., admission for respiratory distress, conjunctivitis, sepsis) via maternal interviews and patient medical records. Should a mother-infant pair not present for a scheduled 6-week follow-up visit, research staff make repeated attempts to contact her to attend clinic. If neonatal mortality is identified, a verbal autopsy is performed, and cause of death is confirmed via medical records. A study supervisor will perform weekly reviews to ensure data completeness and validity; discrepancies are resolved using information that is collected from the delivery register. If the swab taken from the mother at the post-natal visit is positive for one of the STIs, the infant’s swabs are also tested and treatment is given where needed at the 6-week follow-up visit.

Finally, the Reach-Effectiveness-Adoption-Implementation-Maintenance (RE-AIM) model is used as a conceptual framework [46–48] to guide the collection of valuable information during this effectiveness trial. A mixed methods approach is used to collect process measures such as recruitment rates, refusal characteristics, perceived and experienced barriers and facilitators to optimal implementation, intervention costs, impact of intervention on patient outcomes, and perceived health system readiness to implement our interventions, and assess modifications that can be made to maximize future implementation success (Table 2).

**Table 2:** Process evaluation

Element	Questions	Measures	Data sources/tools
Reach	1) What % of eligible patients consented to receive the intervention?	1) Recruitment rates	1) Enrolment tracking sheets
	2) Do those that consent differ significantly from those that do not?	2) Socio-demographics of all eligible participants stratified by consent/refused	2) Enrolment tracking sheets
Effectiveness	What is the effect of the intervention on patient outcomes?	Main study outcomes comparing interventions and control	Study datasets
Adoption	1) What are the main barriers/facilitators to adopting the intervention?	1) Perceptions of research/clinic staff, facility management, National Health Laboratory Service (NHLS) & National Dept. of Health (NDoH)	1) Staff observational logs and post-intervention interviews
	2) What systems need to be in place for the health system to adopt intervention?		2) Post-intervention interviews clinic and national stakeholders
Implementation	1) What does the intervention cost?	1) Cost/Cost-effectiveness data	1) Study datasets
	2) What support and tools are needed for consistent delivery of intervention?	2) Perceptions of study and clinic staff, NHLS and NDoH	2) Post-intervention interviews w/ clinic and national stakeholders
Maintenance	1) What resources will be needed for the intervention to be sustainable?	1) Perceptions of research staff, facility managers, NHLS and NDoH	1) Research staff observation logs, post-intervention interviews
	2) What adaptions are needed to integrate intervention into current practices?		2) Post-intervention interviews clinic and national stakeholders

#### Aim 2

While Aim 1 will determine the efficacy of our screening interventions in improving birth outcomes for pregnant women, Aim 2 will determine whether the monetary costs of our interventions are cost-saving or cost-effective. This analysis will take into account the costs of each intervention, costs averted, and the overall cost-effectiveness using a societal (government provider and patient) perspective.

##### The provider perspective

The costing will establish the utilization of health services (e.g., diagnostic and treatment visits), diagnostic tests, and medication from trial data specific to each arm. Within a decision analytic modeling framework, those utilization estimates will be multiplied by the full economic or unit cost of each service, diagnostic test, or medicine. Unit costs will be computed using a micro costing with bottom-up and step-down allocation approaches, as appropriate. For example, for diagnostic visits, bottom-up costing captures staff time for diagnosis, while step-down approaches are used to apportion shared costs within the facility such as managerial, clerical, cleaning and security staff, and utilities. For diagnostic tests, bottom-up costing will be used to capture the costs of the test cartridges and GeneXpert machines (appropriately annuitized). Similarly, the costing of adverse pregnancy or birth outcomes entails the bottom-up costing of clinical staff, infrastructure and equipment within the facility where care is provided (e.g., neonatal intensive care unit), together with a step-down allocation of shared costs such as overheads within the hospital. When valuing resources within the cost analysis that are paid from the research budget, we will use routine public sector “prices” for staff and medication and will seek to cost GeneXpert machines and cartridges at a level commensurate with a potential public sector scale-up. Care will be taken to exclude any costs that are incurred only as part of research activities.

##### The patient perspective

We will collect demographic, socio-economic, patient cost, and income data via the REDCAP questionnaire. Data will be collected at each assessment unless the variable is expected to stay constant over the study period (e.g., educational status). Socio-economic status will be computed via a multiple correspondence analysis on household type, assets, and access to services following established methodology [49, 50]. Patient costs will include transport costs, opportunity costs of travel, waiting and visit times, out-of-pocket payments, and any income lost while seeking care. To increase response rates, we will use a categorical approach to collecting data on income and transform this into a quantitative variable using a regression methodology, where income can be predicted as a function of demographic and socioeconomic status [49]. The opportunity cost of time will be valued using wages/salary earnings foregone [51]. In order to value these costs equitably, the mean income reported across all participants at the baseline assessment will be used as a proxy of this opportunity cost. In contrast, time, travel and user fee costs will be compared to the respondent’s personal income in order to assess the share of income spent on these costs.

### Specimen collection

**Fig. 3:**
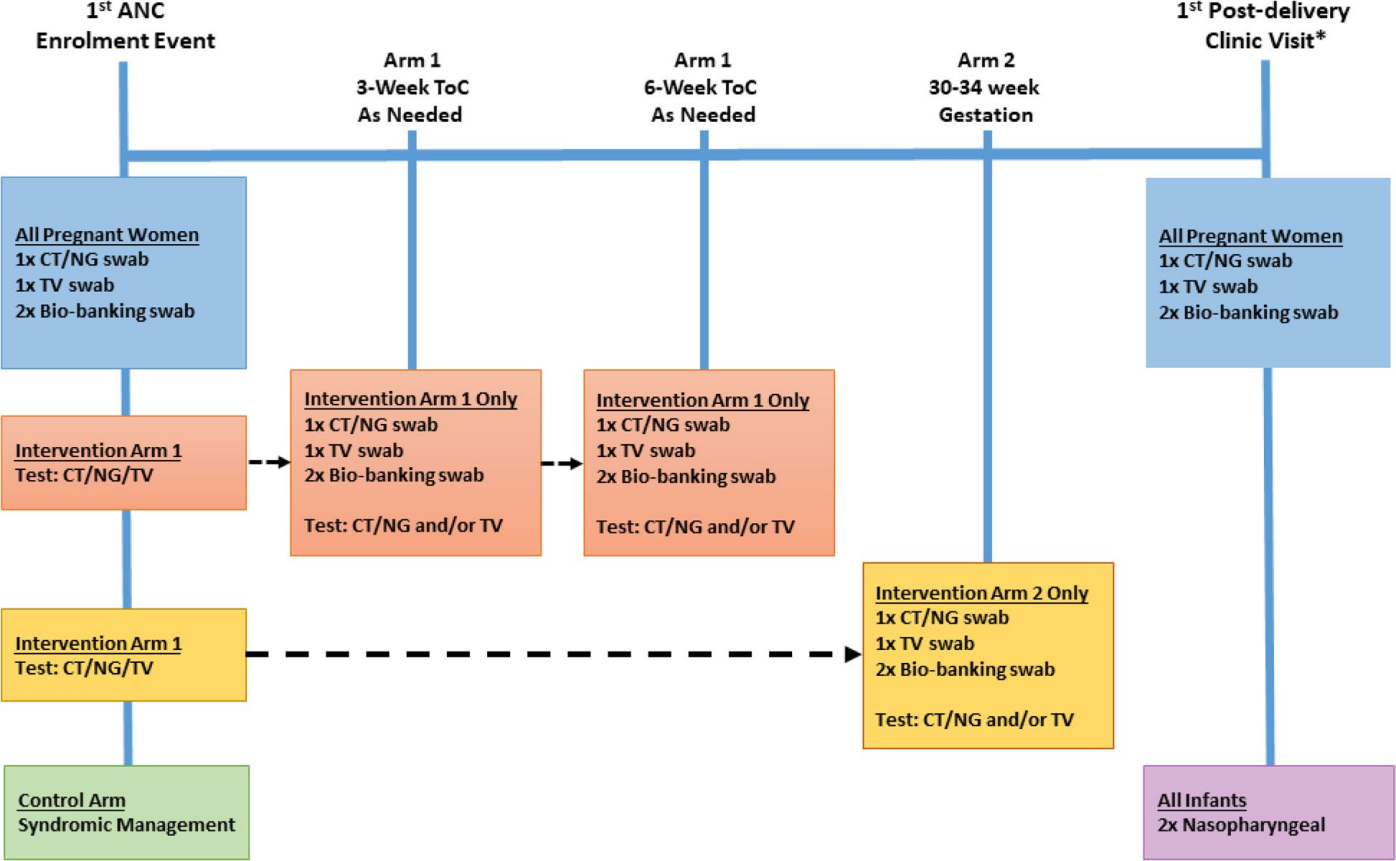
Specimen collection and tests performed at each study time point. *Post-delivery vaginal and NP swabs will be batch tested using Xpert® CT/NG and Xpert® TV assays at the end of the study

Study nurses will collect four vaginal swab specimens: 2 swabs for STI testing (one for CT/NG testing; one for TV testing) and 2 swabs for bio-banking for future research (Fig. 3). The GeneXpert Vaginal/ Endocervical Specimen Collection kit [Cepheid, Sunnyvale, CA] is used for vaginal swab specimen collection. For specimen bio-banking, participants use a dry FLOQswab® [COPAN, Murrieta, CA] for specimen collection, with subsequent storage in a sterile tube. Vaginal pH of participants is measured on pH strips using vaginal secretions collected from a swab used for STI testing; pH strips are interpreted using the manufacturer’s chart [52]. If a participant is not comfortable with providing nurse-collected vaginal swabs specimens, they are given the option to provide a urine specimen for testing.

During the first postnatal visit (typically 3–6 days post clinic discharge), four vaginal swabs are collected from all post-partum women to provide a proxy for STI at time of delivery: 2 swabs for STI batch testing (one for CT/NG testing; one for TV testing) and 2 swabs for bio-banking for future research. In addition, two nasopharyngeal swabs and two conjunctival swab specimens are collected from all infants for STI testing should their mother test positive for an STI. Specimens are labeled with random specimen identifying numbers that link to participant IDs. Specimens are transported to the University of Pretoria and stored as described below. One vaginal and one infant swab (when the maternal swab is tested positive) are batch tested using Xpert® CT/NG and Xpert® TV assays. Test results are used for treatment, as needed, at the 6-week infant immunization visit.

Staff handle specimens and label with a unique study barcode to link a participant’s STI test results, medical chart, and study questionnaire data. Specimens are stored at 2–8 °C and transported to the University of Pretoria on a bi-weekly basis according to Good Laboratory Practice. Specimens are flash frozen and stored at − 80 °C for bio-banking.

### Diagnostic testing

Vaginal specimens collected from participants are tested for CT, NG, and TV using the Xpert® CT/NG and Xpert® TV assays [Cepheid, Sunnyvale, CA]. Trained STI test counselors and research nurses conduct the point-of-care testing at each of the clinical sites. Once collected, research staff follow test kit instructions for swab preparation and testing. Xpert® CT/NG provides 90-min detection and differentiation of CT and NG, while Xpert® TV provides 60-min detection of TV, with early termination for positive results after 40 min; both test cartridges have high sensitivity and specificity [52] and function well in resource-constrained environments and clinical settings. Each Xpert test includes a sample processing control to ensure correct cell lysis/DNA extraction of the sample, a sample adequacy control to ensure adequate human DNA in the specimen, and a probe check control, which monitors reagent rehydration, reaction-tube filling, probe integrity, and dye stability. If testing cannot be conducted due to power failures, errors, or testing delays, specimens are stored at 2–4 °C in a secure storage area for up to 24 h until tested.

Should a participant test positive for NG, dependent on the clinical history, a specimen for culture and susceptibility testing is obtained.

### Reporting and treatment

Study hired STI test counselors report all test results to the ANC research nurses, who provide test results’ notification to study participants. Women with negative test results are provided safe sex counseling. Women testing positive for any STI are provided treatment per South African National STI treatment protocols, counseled on safe disclosure to their partners, assessed for potential intimate partner violence related to disclosure, and given a partner notification slip [53, 54]. During antenatal care, Arm 1 and 2 participants are provided same day results and immediate treatment; those with a positive test-of-cure at their 3-week visit (Arm 1) are again provided appropriate treatment. Post-delivery, all study participants are provided results and treatment at their next routine post-natal baby wellness visit. For women assessed via syndromic management, the study makes use of approved first-line syndromic treatment regimens for STIs as per CDC and South African guidelines: for CT, participants testing positive receive two 500 mg tablets of oral azithromycin; for NG, participants testing positive receive 250 mg of intramuscular ceftriaxone and two 500 mg tablets of oral azithromycin; and for TV, participants testing positive receive 400 mg metronidazole twice a day for 7 days.

### Data management

Each potential participant screened for eligibility is assigned a unique survey identification number by REDCap. This does not include any personal identifying information. Data are stored in a secure, password-protected, web-based database which is only accessible to authorized project staff. Tablet computers used for interviews and extraction of data from medical records are password protected and are stored securely at study offices. Paper records of participants are kept in lockable filing cabinets at study offices; forms with identifiers are kept separately from demographic, clinical, and other data. Paper records, excluding informed consent forms, only contain unique survey identification numbers. A separate, access controlled, link log database is maintained by the data manager. The link log is stored separately from the rest of the study data and is only accessed when absolutely necessary. Lab case report forms are stored separately from any other documents that contain identifiable information. Qualitative data including audio files and password-protected transcripts are stored on a secure, access-controlled cloud-based database.

Test results and clinical data are directly entered into REDCap. Automated data quality checks and skip patterns are also built into REDCap. STI test counselors and research nurses conduct onsite data quality checks daily under the supervision of a field coordinator. Data administrators conduct data quality checks weekly and flag any inconsistencies for field-based staff to rectify. Data are also checked against hard-copy source documents for consistency. All research study personnel meet weekly to review study enrollment, specimen collection, processing, test turn-around-time, data management, and treatment outcomes. Meetings discuss descriptive study results to date, problems encountered, and remedial actions to be taken. Field-based staff are invited to monthly team meetings to discuss the above and/or take the form of a refresher training where needed to ensure study protocol compliance.

Scheduled and unscheduled data quality inspections are carried out by data quality assurance personnel in order to ensure high data quality standards. The Principal Investigators, or their designee, randomly select 10% of all participant files for inspection every 3 months. An external study monitor will also be consulted at three time points during the study to conduct an external audit of source documents as well as the regulatory study binder. Finally, the study includes a Data Safety and Monitoring Board, which reviews the list of all adverse events that occur at any time during the study and has ultimate ability to terminate the trial should interventions prove to have unacceptable risk. Members of this board will have no direct association with the study nor study sponsors.

### Statistical analysis

#### Aim 1

Data will be analyzed using R [R Foundation for Statistical Computing, Vienna, Austria] and SAS 9.4 [Cary, North Carolina]. Participant demographic and clinical characteristics will be described per study arm using proportions (categorical variables), as well as measures of central tendency (sample mean, sample median) and dispersion (sample variance, interquartile range) for continuous variables. Outcome difference among treatment arms will be assessed for statistical significance using chi-square tests and logistic regression models for categorical/binary outcomes. Analysis of variance (ANOVA) and multiple linear regression models will be used for continuous outcomes. Normal probability plots will be used to access the normality assumption for ANOVA and multiple linear regression models. If the normality assumption appears violated, non-parametric procedures will be utilized.

Within Arm 1, we will use 95% confidence intervals for proportions to estimate the percent of women with a negative test-of-cure, but with an STI at birth outcome. These confidence intervals, calculated by HIV status as well as pooled across HIV status, will allow an estimation of the percent of STI prevalence at birth outcome which is due to new infections between ANC visits. Within Arm 2, a logistic regression model will be developed utilizing incident STIs (negative at first ANC visit, positive at 30–34-week ANC) to determine if there is an optimum gestational age at which a second STI screening would be most beneficial or if the data indicates a steady probability across gestational ages. All analyses will be conducted using intent-to-treat principles. Overall type I error rate will be set at 0.05; for multiple comparisons among study arms, type I error will be set to a Bonferroni-corrected type I error of 0.01667. We will use multiple imputation of missing data when missing values exceed 10% and will conduct sensitivity analyses to determine how imputed data affects the study results.

Primary outcomes to be compared among study arms, adjusted/controlling for HIV-infection status include (1) frequency of adverse birth outcomes and (2) change in STI prevalence between baseline (first ANC visit) and birth outcome. We will calculate the change in CT, NG, and TV prevalence by subtracting the prevalence at delivery from the prevalence at baseline. We will use generalized estimating equations to test for variation among study arms with regard to change in prevalence of CT/NG/TV between baseline and delivery, adjusting for potential effect modifiers and confounding variables.

We will also investigate four secondary outcomes: (1) prevalence and risk factors for CT, NG, and TV colonization in neonates controlling for HIV status; (2) among mothers, the prevalence and risk factors for STI infection at birth outcome; (3) factors associated with STIs at first ANC; and (4) process evaluation measures as described in Table 2. Finally, we have two exploratory outcomes for Aim 1: (1) the type and frequency of adverse birth outcomes as a function of STI and HIV status and (2) infant outcomes, including pneumonia and neonatal conjunctivitis, at 6 weeks.

We will analyze the process evaluation qualitative data using aspects of deductive analysis that consider the RE-AIM framework through the creation of initial a priori codes. Data coding and analysis will be an iterative and interactive process. Interview transcripts will be read to increase familiarity with data. A priori and emergent codes will be assigned. Transcripts will be re-read to create pattern codes that connect subsequent concepts under larger headings. Consistent patterns in meaning, concepts, and themes across interviews will be identified, and data matrices created as visual representations of findings [48–50]. We will also examine any differences based on stakeholder type (i.e., study staff, non-study clinic staff, National Health Laboratory Service and Health Department) to identify unique viewpoints. Coding and analytic activities will be discussed during qualitative data analysis meetings; discrepancies in coding and interpretation will be resolved through consensus.

Finally, we will use a predictive modeling approach to develop a STI risk calculator, for any STI as well as separately for CT, NG, and TV [55]. To assure model utility, we will select variables that are readily available to clinicians a priori. Model building will utilize tenfold cross validation where the data is randomly divided into 10 datasets. For each model fitting iteration, 9 of the datasets will be used to fit the model. This resulting model will then be used to predict outcomes in the 10th dataset. The final model will be a weighted average of the models observed in each of the 10 cross-validation steps. Weights will be assigned based upon observed degree of fit with models exhibiting higher degree of fit (better prediction) receiving higher weights.

#### Aim 2

We will build a decision analytic model to estimate costs and outcomes for each study arm and perspective (provider/patient). For DALY calculations, years of life lost are the difference between age at death and average South African life-expectancy for that age; years of life with disability and disability weights will be estimated from the Global Burden of Disease studies [56, 57]. Deterministic sensitivity analyses will assess the impact of key parameter uncertainty (e.g., cost of GeneXpert machines within a scale-up scenario). Probabilistic sensitivity analysis will assess uncertainty around each utilization estimate from the trial [58]. If we find that the costs of Arms 1 and/or 2 exceed the costs of Arm 3, we will compute incremental costs per STI and DALY averted. For value for money determinations, the latter will be compared to a cost-effectiveness threshold based on the estimated marginal productivity of the South African public health care system [59]. For the patient perspective, catastrophic expenditure will be computed by comparing patient costs to income using a variety of thresholds per other South African and low- and middle-income country studies [60].

### Dissemination

As results are available and finalized, they will be analyzed for publication in peer-reviewed scientific journals and presentation at relevant scientific conferences; standard authorship guidelines will be followed, and no professional writers will be used. Results will also be presented, orally and in writing, to local (i.e., Buffalo City Metro District Department of Health), provincial (i.e., Eastern Cape Provincial Department of Health, Eastern Cape Provincial AIDS Council), and key national (i.e., South African National Department of Health, South African National HIV Think Tank) stakeholders. Results will be reported back to study clinic staff, communities and participants, via town hall style meetings and as 1-pager flyers using infographics. Key interim results (i.e., STI prevalence, incidence and pregnancies outcomes), implementation experiences, and lessons learned will be shared with district and provincial stakeholders on a bi-annual basis.

## Discussion

Current WHO STI screening recommendations, especially during pregnancy, leave a large burden of infections undetected and untreated. With the advent of new, rapid, easy-to-use PCR-based “near-patient” or “point-of-care” technology for the diagnosis of STIs [61, 62], the implementation of diagnostic screening in variety of clinical settings is now possible [26, 63–67]. Despite that, optimal models for point-of-care testing, especially during pregnancy, have not been identified. Demonstrating the impact of diagnostic screening and treatment, compared to syndromic management, on birth outcomes will provide critical evidence to update WHO’s syndromic management guidelines during pregnancy.

There are a number of potential unintended consequences to participating in an STI screening study. Partners are an important component in STI (re)infection and treatment. Participants who test positive for an STI are encouraged to disclose to their partner(s); however, as a result of gender and relationship power dynamics, women are often blamed for bringing the infection into the relationship. High rates of intimate partner violence in South Africa may cause women to be scared to disclose their STI status. Disclosure may also have unintended consequences of a social, emotional, physical, and/or financial nature. To mitigate this risk, we have formulated the following:

1. All participants receive counseling as per clinic standards once STI results are available.
2. All participants are counseled on the unintended social risks with STI disclosure.
3. Women testing positive for an STI are counseled on safe disclosure to their partners.
4. Women are given the referral for their partner(s) to present to the clinic.
5. All participants are assessed for potential intimate partner violence related to disclosure.
6. Participants are asked to report any social harms. Appropriate counseling, care and referral are offered depending on the nature of the social harm reported. Any social harms that arise during the study are recorded and reported as per the National Institutes of Health and local IRB guidelines.
7. In the case where issues relating to intimate partner violence or mental health are detected, participants are offered to see the social worker or clinic psychologist. Additional information on organizations that provide intimate partner violence support services (e.g., such as hotlines, local trauma centers) can be extended to participants where needed.

With these protections in place, women who participate in this study benefit from better access to syndromic STI screening, treatment, and diagnostic testing. Better STI management in turn allows for certain health benefits for both the participant and the neonate. Additionally, information learned in this study may help to reduce adverse birth outcomes and assist with efforts to prevent mother-to-child transmission of HIV. Participants found to have persistent STI infections are referred for specialized treatment and care, and clinical notes and/or referrals assist the participant beyond the duration of the study.

In addition to the main randomized-controlled trial, our team will use a cohort design with a nested case–control study to investigate associations between the presence of lower genital tract organisms in pregnancy and adverse pregnancy outcomes, and investigate the relationship between the vaginal microbiome and persistent chlamydial infection in pregnant women. Research suggests that the vaginal microbiota plays a critical role in STI acquisition, persistence, and treatment outcomes. Vaginal community state types with different concentrations of *Lactobacillus (L.)* species are associated with increased risk of acquiring STIs [68–72]. In vitro studies revealed certain vaginal bacteria can inactivate metronidazole [73–75], standard TV treatment, and bacterial vaginosis (BV; Community State Type 4) which influenced TV treatment outcomes in women living with HIV [76]. Vaginal microbiomes dominated by *Lactobacillus crispatus*, *Lactobacillus gasseri*, and *Lactobacillus vaginalis* may inhibit CT elementary bodies [77], while *Lactobacillus iners* may increase the risk and duration of CT infection [78]. These supplemental observations study components will enhance the trial findings and allow our team to further investigate associations between the presence of lower genital tract organisms in pregnancy and adverse pregnancy outcomes, as well as the relationship between the vaginal microbiome and persistent chlamydial infection in pregnant women.

### Trial status

This paper reflects protocol version 0.5, dated 04 October 2021. The trial was first registered on ClinicalTrials.gov on 25 June 2020, with the last update posted 29 June 2021 (NCT04446611). Participant recruitment began 29 March 2021 and is expected to be completed in April 2024.

## Data Availability

The study co-primary investigators (AMM and JDK) will provide the full study protocol, study data, and statistical code upon reasonable request and approval of an appropriate data sharing agreement.

## Abbreviations

ANC: Antenatal care
BV: Bacterial vaginosis
CT: *Chlamydia trachomatis*
DALY: Disability adjusted life year
NG: *Neisseria gonorrhoeae*
PCR: Polymerase chain reaction
STI: Sexually transmitted infection
TV: *Trichomonas vaginalis*
WHO: World Health Organization
